# Unclean cooking fuel use and stillbirth in Ghana: evidence from the 2022 DHS

**DOI:** 10.3389/fgwh.2025.1636924

**Published:** 2025-10-23

**Authors:** Kirstin P. West, Kristin K. Sznajder, Grace Hwang, Hannah E. Sauve, Kedir Teji Roba, Leonard Baatiema, Ernest Kenu, Charles L. Noora, Abraham Tamirat Gizaw, Abebayehu N. Yilma

**Affiliations:** 1 Department of Public Health Sciences, Pennsylvania State University College of Medicine, Hershey, PA, United States; 2 Institute of Energy and the Environment, Pennsylvania State University, University Park, PA, United States; 3 Department of Global Health, University of Washington, Seattle, WA, United States; 4 Department of Epidemiology, School of Public Health, Boston University, Boston, MA, United States; 5 Department of Biobehavioral Health, Pennsylvania State University, College of Health & Human Development, University Park, PA, United States; 6 College of Health and Medical Sciences, Haramaya University, Dire Dawa, Ethiopia; 7 Department of Health Policy Planning and Management, College of Health Sciences, School of Public Health, University of Ghana, Legon, Ghana; 8 Department of Epidemiology and Disease Control, College of Health Science, School of Public Health, University of Ghana, Legon, Ghana; 9 Faculty of Public Health, Department of Health, Behavior and Society, Institute of Health, Jimma University, Jimma, Ethiopia

**Keywords:** stillbirth, unclean cooking fuel, household air pollution, maternal health, environmental risk factors, Ghana, demographic and health survey

## Abstract

**Introduction:**

Stillbirth remains a major public health issue in low- and middle-income countries (LMICs). Ghana's 2021 stillbirth rate (21.4 per 1,000 births) exceeds the United Nations Sustainable Development Goal (SDG) target of 12 per 1,000 births by 2030. Unclean household cooking fuels have been associated with adverse pregnancy outcomes, including stillbirth. In Ghana, women conduct about 64% of household cooking, often in poorly ventilated settings with particulate levels above World Health Organization (WHO) guidelines. We assessed the association between household cooking fuel type and stillbirth among Ghanaian women.

**Methods:**

We conducted a cross-sectional analysis using data from the 2022 Ghana Demographic and Health Survey. The sample included 10,654 women aged 15–49 years with ≥1 recorded pregnancy. The primary exposure was household cooking fuel (clean vs. unclean per WHO guidelines). Outcomes were (1) stillbirth, defined as fetal loss at ≥7 months’ gestation, and (2) stillbirth rate per 1,000 total births. Survey-weighted bivariate screening (*p* < 0.05) identified candidate covariates for inclusion in multivariable, survey-weighted logistic regression models. Adjusted odds ratios (AORs) and 95% confidence intervals (CIs) were reported. Given the cross-sectional design, estimates reflect associations, not causation.

**Results:**

The overall stillbirth rate was 15.85 per 1,000 births. Unclean cooking fuel use was associated with 44% higher odds of stillbirth (AOR: 1.44; 95% CI: 1.05–1.99; *p* = 0.0258). Other factors associated with higher odds were age ≥30 years (AOR: 2.17; 95% CI: 1.59–2.95; *p* < 0.001), moderate-to-poor health (AOR: 1.78; 95% CI: 1.39–2.28; *p* < 0.001), and alcohol consumption (AOR: 1.43; 95% CI: 1.06–1.93; *p* = 0.0195).

**Discussion:**

In this nationally representative sample, unclean cooking fuel use was associated with increased odds of stillbirth. Expanding access to clean fuels and leveraging antenatal care services for culturally responsive clean-energy counseling may help reduce stillbirth risk. Prospective studies with exposure monitoring are needed to establish temporality.

## 1 Introduction

Stillbirth remains a major public health concern globally, particularly in low- and middle-income countries (LMICs) where the burden of fetal loss and early neonatal death is disproportionately high (1–5). Although the global stillbirth rate declined from 21.4 to 13.9 per 1,000 total births between 2000 and 2019, this 35% reduction has lagged behind more substantial declines observed in maternal and under-five mortality indicators during the same period (1, 6). Stillbirth prevention has historically received less attention than other mortality indicators in national health agendas and global reporting frameworks (7, 8). Sustainable Development Goal 3.2 (SDG 3.2) calls for reducing stillbirths to 12 per 1,000 total births by 2030; many LMICs remain off track to meet this target (2, 9, 10). Sub-Saharan Africa and South Asia collectively account for 84% of all stillbirths globally (11), and West and Central Africa experience rates up to eight times higher than those in Western Europe (6). In Ghana, the stillbirth rate remains high, with recent estimates of 21.4 per 1,000 total births in 2021 (12, 13).

Many stillbirths are preventable. Recent efforts to reduce them have focused primarily on clinical interventions, such as skilled birth attendance, improved antenatal care, and emergency obstetric services (14–17). In parallel, environmental exposures are increasingly recognized as potential contributors, with numerous studies reporting associations (18, 19). One emerging area of interest is household cooking fuels and their relation to maternal and neonatal health outcomes (18, 19).

**Figure 1:**
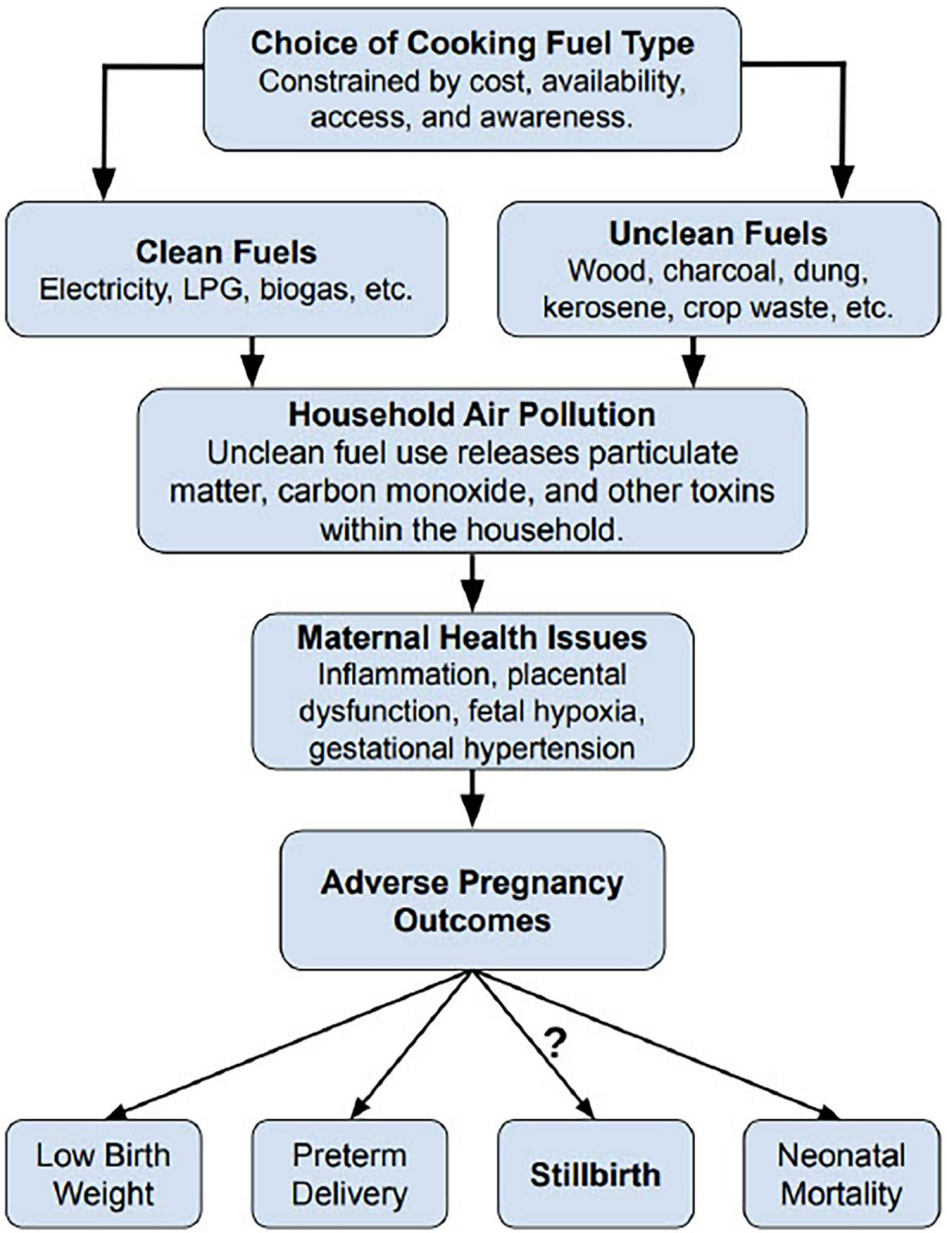
Conceptual framework linking cooking fuel type to stillbirth in Ghana. Fuel choice —often shaped by socioeconomic and contextual factors— may be associated with use of unclean cooking fuels. Combustion of these fuels emits harmful pollutants, notably fine particulate matter (PM_2.5_) and carbon monoxide (CO). PM_2.5_ consists of inhalable particles ≤2.5 μm that can reach the alveoli and enter the bloodstream. CO is a colorless, odorless gas that binds hemoglobin and can reduce oxygen delivery to the placenta and fetus. These pollutants contribute to household air pollution and prolonged exposure has been associated with adverse pregnancy outcomes. Stillbirth remains an underexplored outcome along this hypothesized pathway in Ghana.

Unclean cooking fuels —such as firewood, charcoal, animal dung, and crop residues— are widely used in Ghana, particularly in rural and low-income households (20–22). Incomplete combustion of these fuels during cooking produces high concentrations of indoor air pollutants, like fine particulate matter (PM_2.5_), carbon monoxide (CO), and nitrogen oxides (NOx), often at levels exceeding World Health Organization (WHO) safety guidelines (18, 22–26). Repeated exposure to these particulate emissions has been associated with hypertensive disorders in pregnancy, placental dysfunction, and fetal hypoxia— conditions that are established risk factors for stillbirth (18, 22–26) (Figure 1).

Although Ghana has launched national programs to promote clean cooking technologies [e.g., liquified petroleum gas (LPG) stoves] (27), adoption remains limited because of structural and cultural barriers (21). Over 80% of Ghanaian households still rely on traditional solid fuels; women perform nearly 64% of the household cooking, often in poorly ventilated settings (20, 21). Barriers to clean fuel adoption include high up-front costs for LPG stoves, inconsistent fuel supply chains, limited public awareness, and cultural preferences for traditional cooking methods (21, 28–30).

While the health consequences of household air pollution from unclean fuels are well documented for outcomes such as respiratory illness, low birth weight, and under-five mortality, few studies have examined stillbirth as a primary outcome—even fewer within Ghana (22, 23, 31). Much of the available literature on cooking fuel and stillbirth draws from older or regional datasets, limiting generalizability to Ghana's evolving social, economic, and policy landscape (32). As stillbirth prevention increasingly becomes a policy priority in Ghana, understanding the role of modifiable environmental exposures like household cooking fuel is critical (27, 32, 33).

To address this identified gap, the current study analyzed newly released data from the 2022 Ghana Demographic and Health Survey (DHS) to assess whether unclean cooking fuel use was independently associated with higher odds of stillbirth among Ghanaian women. Given Ghana's rapid development alongside persistent health inequities, these findings may inform national public health strategies and broader global efforts to reduce preventable fetal deaths. By centering environmental exposures within the broader maternal-health agenda, this study underscores the importance of interdisciplinary, multisectoral approaches to stillbirth prevention.

## 2 Materials and methods

### 2.1 Participants

This study used individual-level data from the 2022 Ghana DHS, a cross-sectional, nationally representative survey that collects information on demographic, maternal, and child health indicators (34). The survey employed a stratified two-stage sampling design to select 18,450 households across 618 clusters in Ghana, yielding interviews with 15,014 women aged 15–49 years. Our analytic sample comprised de facto Ghanaian women of reproductive age in the Individual Recode file (GHIR8BFL.SAV). To focus the analysis on pregnancy-related outcomes, we further restricted the sample to women with at ≥1 recorded pregnancy, resulting in a final weighted sample of 10,654 women. Ethical approval for the 2022 Ghana DHS was obtained by the Ghana Health Service Ethical Review Committee and the Institutional Review Board of Inner-City Fund (ICF) International. Written informed consent was obtained from all participants at the time of data collection (34).

### 2.2 Stillbirth definition(s)

The primary outcome was stillbirth, defined as fetal loss at ≥7 months (≥28 weeks) of gestation, consistent with the WHO definition (35). In the DHS Women's Questionnaire, pregnancy duration was recorded in completed months based on maternal recall (34). We derived a binary variable indicating whether a woman had ever experienced a stillbirth: coded as “1” if any reported pregnancy lasted ≥7 months and resulted in a fetus classified as “born dead,” and “0” otherwise. This outcome represents the prevalence of stillbirth among all women in the sample. We also calculated each woman's stillbirth rate, defined as the number of stillbirths divided by the sum of live births and stillbirths, scaled per 1,000 births. Data were available for up to 20 recorded pregnancies per woman.

### 2.3 Type of cooking fuel

The primary exposure was the type of household cooking fuel. In the 2022 Ghana DHS Household Questionnaire, the respondent is asked a single, mutually exclusive question: “What type of fuel does your household mainly use for cooking?” (34). Following WHO guidance, responses were recoded to a binary indicator for cooking fuel type — clean vs. unclean (36). Clean fuels included electricity, LPG, natural gas, alcohol (i.e., ethanol), and biogas. Unclean fuels (e.g., solid fuels) included kerosene, coal or lignite, charcoal, wood, straw, shrubs, grass, agricultural crop waste, processed biomass pellets, garbage, plastic, sawdust, and animal dung.

Women missing data on their primary cooking fuel type or with unclassifiable fuel types (*n* = 200; 1.9% of total weighted sample) were coded as missing. To assess differential missingness, design-adjusted chi-squared tests were used. Missingness was not significantly associated with place of residence, educational status, wealth, drinking water source, radio use, age at first birth, contraceptive use, body mass index (BMI), self-reported health status, alcohol consumption, or sexually transmitted infection (STI) history (all *p* > 0.05). Missing cooking fuel type data were slightly more common among younger women (*p* < 0.001) and among those who had ever experienced a stillbirth (*p* < 0.01). Complete-case estimates are presented; these limitations are noted in the Discussion.

### 2.4 Other variables

Sociodemographic covariates included maternal age (15–29 years vs. ≥30 years), place of residence (urban vs. rural), education status (no schooling to secondary vs. higher education), and the DHS wealth index, a composite indicator reflecting household ownership of assets and access to amenities (e.g., poor, middle, rich).

Household-level covariates included the primary source of drinking water, classified as improved (e.g., piped water, covered boreholes, protected wells, rainwater collection, bottled water) or unimproved (e.g., unprotected wells, surface water sources such as rivers and lakes). Classifications were consistent with WHO and United Nations Children's Fund (WHO/UNICEF) Joint Monitoring Program definitions (37, 38). Exposure to media was approximated by radio usage, classified dichotomously (listens vs. does not listen to the radio).

Maternal characteristics comprised age at first birth (<15–24 years vs. ≥25 years), current contraceptive use (yes vs. no), and BMI (kg/m^2^). Because BMI had substantial missingness (49.3%), we examined correlates of missingness with design-adjusted chi-squared tests. BMI missingness was not associated with the primary exposure (cooking fuel type, *p* = 0.81) and outcome (ever-stillbirth, *p* = 0.34), nor with maternal age, education, residence, self-reported health status, alcohol use, or other household-level factors (all *p* > 0.05). Although associations were noted with wealth index (*p* = 0.04; <4 percentage point difference across groups) and contraceptive use (*p* = 0.001; < 4 percentage point difference across groups), the absence of strong systematic patterns suggested data were plausibly missing at random. Therefore, missing BMI values were imputed using multiple imputation with predictive mean matching (*m* = 5) via the “mice” package in R. Variables included as predictors in the BMI imputation model were maternal age, education, wealth index, parity, source of drinking water, smoking status, and the survey sample weight to account for complex survey design. Imputed BMI was used in descriptive summaries; BMI was not included in the final multivariable model.

Smoking status was based on women's current use of tobacco products and categorized as “smokes” (e.g., currently uses tobacco) or “does not smoke” (e.g., no current tobacco use). Alcohol consumption was measured based on the respondents' reported frequency of alcohol use in the past month. This variable was dichotomized into two categories: “consumes alcohol,” which included women who reported drinking alcohol at any frequency within the past month, vs. “does not drink.” Self-reported health status reflected respondents' personal assessment of their overall health. Women rated their health status using DHS categories: “very good,” “good,” “moderate,” “bad,” or “very bad.” These were subsequently collapsed into two groups for analysis, specifically “good” (including “very good” and “good”) and “moderate-to-bad” (including “moderate,” “bad,” and “very bad”). Presence of any STI in the past year was self-reported (e.g., “yes,” “no,” or “don't know,” with the latter treated as missing).

Maternal healthcare variables —number of antenatal care (ANC) visits for the most recent pregnancy, place of delivery (home, public facility, private/other), and whether the woman had a cesarean section— were summarized descriptively only. In the DHS, these items are collected only for pregnancies within 5 years preceding the survey; consequently, they were missing for >50% of our analytic sample. Because this missingness is determined by survey design and depends on the recency of pregnancies, it is not missing at random and it is directly related to our outcome (e.g., ever-stillbirth, stillbirth rate). Multiple imputation was therefore not inappropriate. To avoid selection bias from conditioning on the five-year pregnancy subcohort, we excluded these variables from bivariate screening and multivariable models.

To ensure model reliability and sufficient statistical power, all candidate covariates were assessed for minimum sample sizes and event counts before the bivariate analyses. Variables with fewer than 100 observations or less than 10% representation in the unweighted sample were collapsed into broader categories. Only covariates that met both theoretical relevance as potential confounders and minimum case count thresholds (≥100 observations and ≥10 stillbirth events) were carried forward into the survey-weighted bivariate analyses. Statistically significant covariates (*p* < 0.05) were retained for inclusion in the multivariable model. Four variables met these criteria and were retained: maternal age (15–29 years vs. ≥30 years), cooking fuel type (clean vs. unclean), self-reported health status (good vs. moderate-to-poor), and alcohol use (no use vs. current use). Final model diagnostics confirmed no convergence issues or inflated standard errors.

### 2.5 Statistical analysis

The DHS employs a complex sampling design involving stratification, clustering, and sampling weights (34). To account for this design in our analyses, survey weights were normalized, and clusters and strata were incorporated into a survey design object using the survey package in R Studio. All estimates were weighted and design-adjusted.

Weighted descriptive statistics were calculated for the primary exposure and covariates (Table 1). Univariable logistic regression estimated crude associations between each covariate and the outcome of ever-stillbirth. All models were weighted and specified using the svyglm() function with a quasibinomial family. Weighted odds ratios (ORs) and 95% confidence intervals (CIs) were reported.

**Table 1:** Weighted sample characteristics of women aged 15–49 with background variables (*N* = 10,654).

Characteristic	Overall *N* = 10,654
Type of cooking fuel, *n* = 10,454, missing = 200	
Clean	2,610 (24.5)
Unclean/Solid	7,844 (73.6)
Maternal age (in years), *n* = 10,654, missing = 0	33.6 (8.3)
Maternal age category (in years), *n* = 10,654, missing = 0	
15–29	3,662 (34.4)
30+	6,992 (65.6)
Place of residence, *n* = 10,654, missing = 0	
Urban	5,791 (54.4)
Rural	4,863 (45.6)
Education status, *n* = 10,654, missing = 0	
No schooling to secondary	9,760 (91.6)
Higher education	894 (8.4)
Wealth index, *n* = 10,654, missing = 0	
Rich	4,488 (42.1)
Middle	2,217 (20.8)
Poor	3,949 (37.1)
Source of drinking water, *n* = 10,476, missing = 178	
Improved water source	9,317 (87.5)
Unimproved water source	1,159 (10.9)
Use of radio, *n* = 10,654, missing = 0	
Yes, Listens to the radio	7,126 (66.9)
No, Does not listen to the radio	3,528 (33.1)
Age at first birth (in years), *n* = 10,160, missing = 494	
<15–24	8,173 (76.7)
25+	1,987 (18.7)
Use of contraceptives, *n* = 10,654, missing = 0	
No, Currently not using	6,827 (64.1)
Yes, Currently using	3,827 (35.9)
Body mass index (in kg/m^2^), *n* = 10,632, missing = 22	26.1 (5.6)
Body mass index category (in kg/m^2^), *n* = 10,632, missing = 22	
<18–25	5,176 (48.6)
>25	5,456 (51.2)
Self-reported health status, *n* = 10,654, missing = 0	
Good	8,069 (75.7)
Moderate to bad	2,585 (24.3)
Use of alcohol, *n* = 10,654, missing = 0	
No, Does not consume alcohol	9,104 (85.5)
Yes, Consumes alcohol	1,550 (14.5)
# of antenatal care visits, *n* = 4,706, missing = 5,948	
No visits	92 (0.9)
1–4 visits	833 (7.8)
More than 4 visits	3,781 (35.5)
Place of delivery, *n* = 4,706, missing = 5,948	
Home	636 (6.0)
Public	3,554 (33.4)
Private or other	516 (4.8)
Received C-section, *n* = 4,706, missing = 5,948	
Yes	952 (8.9)
No	3,754 (35.2)
Had sexually transmitted infection in past year, *n* = 10,649, missing = 5	
Yes	648 (6.1)
No	10,001 (93.9)

The table presents weighted distributions of sociodemographic, household, and maternal health variables. Categorical variables are shown as *N* (%); continuous variables are reported as mean (standard deviation). Sample sizes (*n*) and missing values per covariate noted.

Bivariate analyses were conducted to examine unadjusted associations between stillbirth and covariates hypothesized to be linked to both cooking fuel use and stillbirth (Table 2). For each covariate level, two outcome measures were reported: (1) the weighted prevalence of ever-stillbirth, and (2) the stillbirth rate per 1,000 total births (Table 2). The first metric reflects the weighted proportion of women who had ever experienced a stillbirth within each level of the covariate. Meanwhile, the second metric captures the intensity of stillbirths relative to the total number of pregnancies. We used design-adjusted chi-square tests to assess whether stillbirth significantly differed across covariate levels. Variables with a *p* < 0.05 were retained for inclusion in the final multivariable logistic regression model.

**Table 2:** Weighted bivariate analysis of stillbirth outcomes stratified by covariates among Ghanaian women aged 15–49.

Variable	Level	*n*	Stillbirths (w%)	Stillbirths per 1,000	*P* value
Maternal age	15–29	3,662	2.66	16.93	<0.001 ***
	30+	6,992	5.80	15.32	
Place of residence	Urban	5,791	4.27	15.26	0.0597
	Rural	4,863	5.26	16.41	
Education status	No schooling to secondary	9,760	4.87	16.08	0.1097
	Higher education	894	3.15	13.23	
Wealth index	Rich	4,488	4.23	16.03	0.1894
	Middle	2,217	4.56	14.35	
	Poor	3,949	5.37	16.46	
Type of cooking fuel	Clean	2,610	3.64	14.89	0.023 *
	Unclean/solid	7,844	5.17	16.41	
Source of drinking water	Improved water source	9,317	4.73	16.13	0.5457
	Unimproved water source	1,159	5.17	15.02	
Use of radio	Yes, listens	7,126	4.44	15.97	0.1441
	No, does not listen	3,528	5.28	15.60	
Age at first birth	15–24	8,173	4.57	11.31	0.8766
	25+	1,987	4.46	14.10	
Use of contraceptives	No, currently not using	6,827	4.63	17.02	0.6602
	Yes, currently using	3,827	4.88	13.74	
Body mass index (kg/m^2^)	<18–25	5,176	4.77	15.94	0.8665
	>25	5,456	4.68	15.78	
Self-reported health status	Good	8,069	3.90	13.48	<0.001 ***
	Moderate to bad	2,585	7.29	23.40	
Use of alcohol	No, does not drink	9,104	4.39	15.01	0.0029 **
	Yes, consumes alcohol	1,550	6.69	20.87	
Had STI in the past year	Yes	648	3.16	17.05	0.1283
	No	10,001	4.82	15.77	

This table presents two complementary stillbirth metrics, stratified by covariate level: (1) survey-weighted percentage of women who reported ever having experienced a stillbirth (prevalence), and (2) survey-weighted stillbirth rate per 1,000 total births. Discrepancies between these measures may reflect differences in fertility patterns across subgroups; that is, certain groups may have fewer women affected overall but higher stillbirth rates among those who are affected. Sample sizes (*n*) are included for each covariate level. Chi-square *p*-values indicate the strength of association between each covariate and binary ever-stillbirth.

Asterisks indicate significance (*p < 0.05; **p < 0.01; ***p < 0.001) from design-adjusted chi-square tests.

A multivariable logistic regression model was fit to assess independent associations with stillbirth using the svyglm() function; a quasibinomial distribution was specified (Table 3). Adjusted odds ratios (AORs) and corresponding 95% confidence intervals (CIs) were obtained by exponentiating model coefficients and standard errors. Statistical significance of covariates was assessed using design-adjusted chi-square tests.

**Table 3:** Unadjusted and adjusted logistic regression model estimates for risk of stillbirth by sample characteristics.

Variable		Unadjusted results			Adjusted results		
		OR	95% CI	*P*-value	OR	95% CI	*P*-value
Maternal age	15–29	1.00			1.00		
	30+	2.25	1.66–3.04	<0.001 ***	2.17	1.59–2.95	<0.001 ***
Cooking fuel type	Clean	1.00			1.00		
	Unclean	1.44	1.05–1.98	0.0237 *	1.44	1.05–1.99	0.0258 *
Self-rated health status	Good	1.00			1.00		
	Moderate to bad	1.94	1.52–2.47	<0.001 ***	1.78	1.39–2.28	<0.001 ***
Use of alcohol	No, does not drink	1.00			1.00		
	Yes, consumes alcohol	1.56	1.16–2.10	<0.0032 **	1.43	1.06–1.93	0.0195 *

Odds ratios (ORs), 95% confidence intervals (CIs), and *p*-values are reported for both unadjusted and adjusted models. Covariates in the adjusted model were selected based on bivariate analyses (*p* < 0.05) and theoretical justification as potential confounders. The adjusted model includes maternal age group, cooking fuel type, self-rated health status, and alcohol use. All covariates remained independently associated with stillbirth in the final adjusted model.

Asterisks indicate significance (*p < 0.05; **p < 0.01; ***p < 0.001) from design-adjusted chi-square tests.

## 3 Results

### 3.1 Sample characteristics

Table 1 presents the weighted demographic, household, and maternal characteristics for 10,654 Ghanaian women aged 15–49 years. The average maternal age was 33.6 years (SD 8.3), with nearly two-thirds of women (65.6%) aged 30 or older. Over half resided in urban areas (54.4%), and most had completed up to a secondary-level education (91.6%). Approximately one-third of participants (37.1%) were in the poorest wealth tertile (Table 1). Most women (73.6%) reported using unclean or solid fuels as their primary cooking fuel; 24.5% used clean fuels. Most respondents lived in households with access to improved drinking water sources (87.5%).

Media exposure was moderate, with 66.9% reporting that they listened to the radio. Nearly four in five women (76.7%) had their first birth between the ages of 15 and 24. Mean BMI was 26.1 kg/m^2^ (SD 5.6), with roughly half of the sample (51.2%) having a BMI over 25 kg/m^2^ (Table 1). Regarding other health variables, 14.5% reported any alcohol use in the past month, 35.9% reported current contraceptive use, and 24.3% rated their health as moderate to poor. A small proportion (6.1%) reported having a sexually transmitted infection (STI) in the past year (Table 1).

Among women with available maternal healthcare data (*n* = 4,706), 35.5% reported more than four ANC visits during their most recent pregnancy (Table 1). Most deliveries occurred in public health facilities (33.4%), while home births (6.0%) and births in private or other settings (4.8%) were less common. Cesarean section deliveries were reported by 8.9% (Table 1).

### 3.2 Bivariate associations with stillbirth

**Figure 2:**
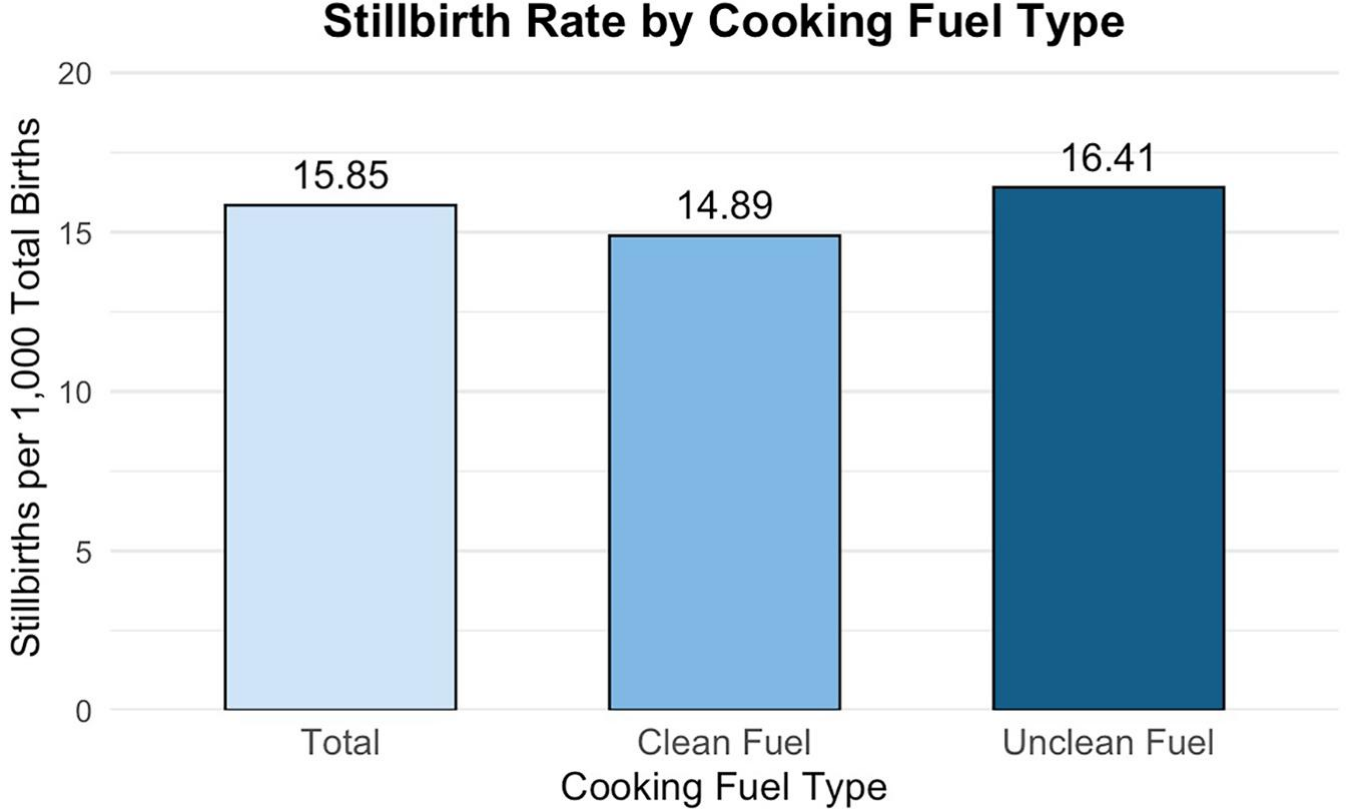
Stillbirths per 1,000 total births by cooking fuel type among Ghanaian women. This figure displays stillbirth rates per 1,000 total births for the overall sample and stratified by cooking fuel type among Ghanaian women aged 15–49 years. Estimates were derived using survey-weighted means to reflect the nationally representative sample. While overall stillbirth rate was 15.85 stillbirths per 1,000 total births, women in households using unclean fuels experienced a significantly higher stillbirth rate (16.41 stillbirths per 1,000 total births) compared to those using clean fuels (14.89 stillbirths per 1,000 total births).

Across the weighted sample, the stillbirth rate was 15.85 per 1,000 births (Figure 2). Table 2 presents both the weighted prevalence of ever experiencing a stillbirth and the stillbirth rate per 1,000 births, stratified by covariate levels. Stillbirth outcomes differed by maternal age, cooking fuel type, self-reported health status, and alcohol use.

Use of unclean cooking fuels was associated with a higher lifetime prevalence of ever having experienced a stillbirth (5.17% vs. 3.64% among women using clean fuels; *p* = 0.023) and a higher stillbirth rate (16.41 vs. 14.89 per 1,000 births).

Age ≥30 years was associated with a higher prevalence of ever having experienced a stillbirth (5.80% vs. 2.66% among women aged 15–29 years; *p* < 0.001). By contrast, the stillbirth rate per 1,000 total births was slightly lower among women aged ≥30 years (15.32 vs. 16.93 per 1,000 births among women aged 15–29 years). This pattern is consistent with higher average parity among older women aged ≥30 years (mean = 4.2 lifetime pregnancies) compared to younger women aged 15–29 years (mean = 1.7 lifetime pregnancies).

Self-rated health was associated with stillbirth outcomes. Women who reported “moderate-to-poor” health had a 7.29% ever-stillbirth prevalence and a stillbirth rate of 23.40 per 1,000 births, compared with 3.90% and 13.48 per 1,000 births among those reporting good health (*p* < 0.001). Alcohol use in the past month was associated with higher ever-stillbirth prevalence (6.69% vs. 4.39% among women who did not consume; *p* = 0.003) and a higher stillbirth rate (20.87 vs. 15.01 stillbirths per 1,000 births among women who did not consume).

### 3.3 Multivariable logistic regression

**Figure 3:**
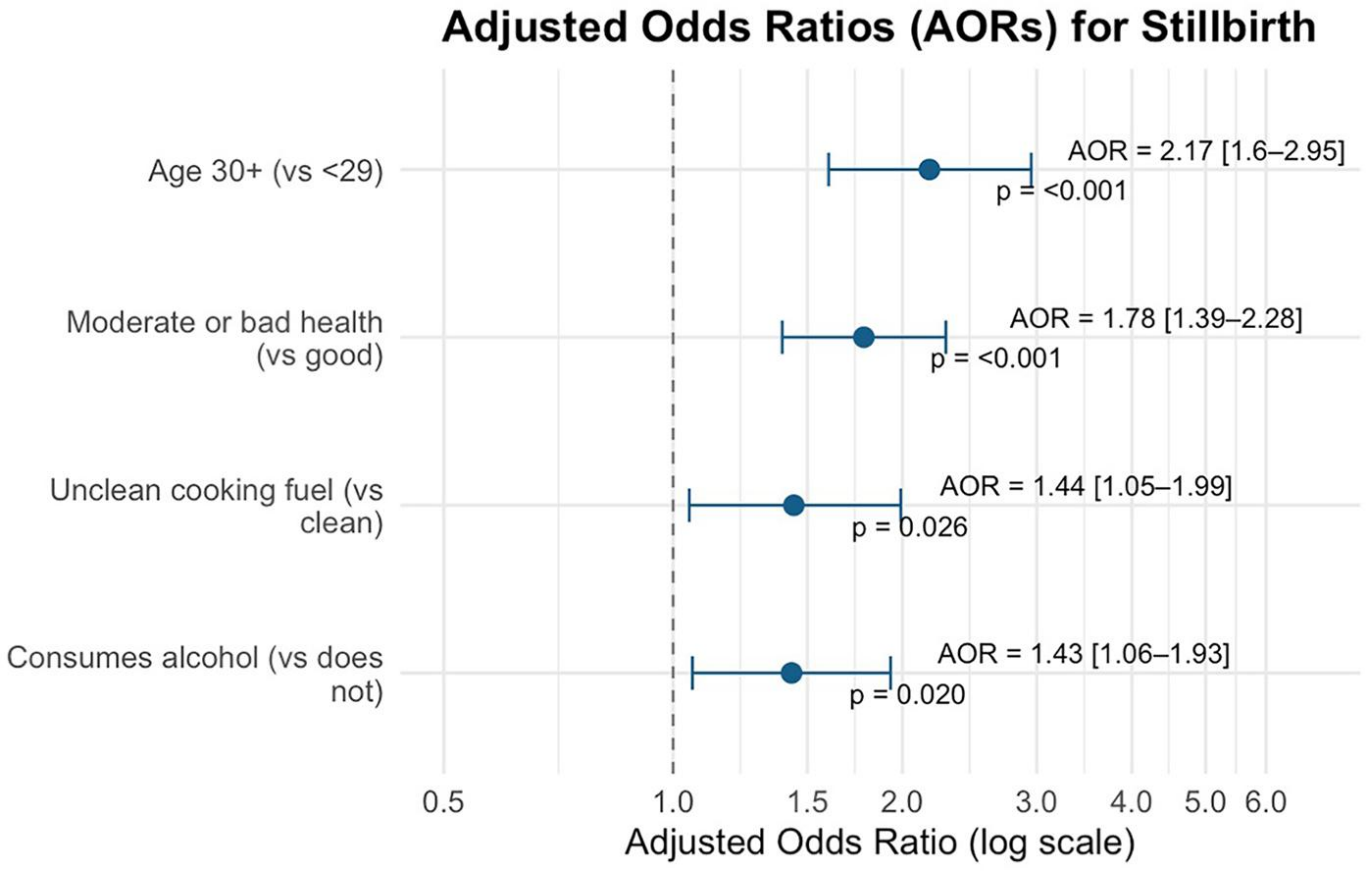
Forest plot of adjusted odds ratios (AORs) for stillbirth. This forest plot presents adjusted odds ratios (AORs) and 95% confidence intervals (CIs) from the final survey-weighted multivariable logistic regression model examining factors associated with ever experiencing a stillbirth. All covariates shown were statistically significant at *p* < 0.05, and had CIs that did not contain the null value. AORs greater than 1 indicate increased odds of stillbirth, while the vertical dashed line marks the null value (AOR=1), indicating no association.

Table 3 and Figure 3 present univariable and adjusted multivariable logistic regression estimates for variables retained after bivariate screening. After adjustment, unclean (vs. clean) cooking fuel use was associated with 44% higher odds of ever having experienced a stillbirth (AOR=1.44; 95% CI: 1.05–1.99; *p* = 0.0258). Maternal age ≥30 years (vs. 15–29 years) was associated with more than twice the odds of ever having experienced a stillbirth (AOR=2.17; 95% CI: 1.59–2.95; *p* < 0.001). Moderate-to-poor self-reported health (vs. good self-reported health) associated with a 78% higher odds of stillbirth (AOR=1.78; 95% CI: 1.39–2.28; *p* < 0.001). Any alcohol use in the past month (vs. none) was associated with a 43% higher odds of ever having experienced a stillbirth (AOR=1.43; 95% CI: 1.06–1.93; *p* = 0.0195).

## 4 Discussion

This study contributes new evidence to the growing literature on environmental exposures and maternal health in Ghana by examining the association between cooking fuel type and stillbirth. Previous research has linked household air pollution from unclean cooking fuel to adverse pregnancy outcomes globally (18, 25, 26, 31); yet, few studies have explored the relationship between cooking fuel type and stillbirth at the population level in Ghana using recent, nationally representative data. We observed that unclean cooking fuel use was associated with 44% higher adjusted odds of ever having experienced a stillbirth (AOR 1.44, 95% CI: 1.05–1.99; *p* = 0.0258). This finding aligns with global and regional evidence associating unclean cooking fuels with adverse pregnancy outcomes (32), as well as with a recent meta-analysis showing a 38% increase in stillbirth odds (39). The stillbirth rate observed in our sample (15.85 per 1,000 total births) remains above the SDG 3.2 target of 12 per 1,000 total births (12). Our results underscore the need to address unclean fuel use as a key, yet under-recognized, contributor to preventable stillbirths.

### 4.1 Biological pathways linking cooking fuel use to stillbirth

The biological mechanisms linking unclean cooking fuels to stillbirth are well-supported by experimental and epidemiological evidence (22, 24, 32, 40–42). Solid fuels such as firewood, charcoal, and crop residues, when burned in poorly ventilated settings, release high concentrations of harmful pollutants. These pollutants—including fine particulate matter (PM_2.5_) and carbon monoxide (CO)—are byproducts of incomplete combustion (22, 25, 26, 43). Such emissions may have direct toxic effects on both maternal and fetal physiology (23, 32, 42).

Inhaled particulate matter can penetrate deep into the lungs, enter the maternal bloodstream, and is thought to trigger systemic inflammation, oxidative stress, and cellular damage to the placenta (24, 44–46). These biological insults may compromise placental function and are associated with fetal growth restriction and intrauterine death (18, 47). Simultaneously, CO binds with high affinity to maternal hemoglobin, potentially reducing oxygen delivery to the placenta and fetus (45, 46). Chronic CO exposure can induce fetal hypoxia and acidemia — conditions strongly associated with stillbirth (26, 46).

Although direct measures of household air pollution (e.g., PM_2.5_ concentration or indoor CO levels) were not available in this dataset, the observed associations between unclean fuel use and stillbirth are consistent with these well-established mechanistic pathways.

### 4.2 Intersecting maternal risk factors and their implications for stillbirth

Beyond fuel use, several maternal characteristics were independently associated with stillbirth in Ghana, including maternal age ≥30 years, poor self-reported health, and alcohol use. Maternal age ≥30 years was associated with more than twice the odds of ever having experienced a stillbirth (AOR=2.17; 95% CI: 1.59–2.95; *p* < 0.001), consistent with broader evidence linking older mothers to increased obstetric risks (48–50). In bivariate analyses, older women (≥30 years) showed a higher lifetime prevalence of ever-stillbirth yet a slightly lower stillbirth rate per 1,000 total births, compared to younger women (15–29 years). This apparent discrepancy is consistent with the outcome metric definitions, whereby prevalence reflects whether a woman has ever experienced a stillbirth across her reproductive lifespan. In contrast, the rate is standardized by the number of births in the denominator. In our sample, women aged ≥30 years had a higher mean parity (4.2 vs. 1.7 births), which increases the denominator and can yield a lower per 1,000 stillbirth rate even when a greater share of women have ever experienced a stillbirth.

Furthermore, the biological plausibility of higher lifetime odds of ever having experienced a stillbirth at older ages is supported, as older women face increased cumulative placental stress, chromosomal abnormalities, hypertensive disorders, along with other obstetric complications over a longer reproductive lifespan (2, 48–50). These patterns may reflect age-related physiologic changes and a greater burden of subclinical placental insufficiency that could interact with household air pollution exposures (51, 52). Longer duration of solid fuel use among older women may also be associated with markers of placental dysfunction and adverse pregnancy outcomes (42, 52). Conversely, the slightly higher per-1,000 stillbirth rate observed among younger women may be consistent with features of biological immaturity, limited socioeconomic resources, reduced access to antenatal care, and less engagement in preventive health behaviors—each of which could plausibly elevate the stillbirth risk in this subgroup (53–56). Because lower socioeconomic status (SES) is associated with both younger maternal age and reliance on solid fuels, the higher stillbirth rate observed among younger women may partly reflect confounding between age, SES, and fuel type, complicating isolation of an age-specific effect independent of fuel exposure (21, 57, 58).

Women who perceived their health as moderate-to-poor were associated with a higher odds of ever having experienced a stillbirth (AOR = 1.78; 95% CI: 1.39–2.28; *p* < 0.001) compared to those reporting good health. While subjective, self-rated health is widely recognized as a valid proxy for overall well-being and may reflect underlying chronic conditions, nutritional status, or psychosocial stress (59, 60). In this context, women with poorer self-reported health may have less physiological reserve to tolerate insults like hypoxia or inflammation resulting from household air pollution (59–61). It is also plausible that women reporting moderate-to-poor health may have higher burdens of chronic conditions like hypertension, anemia, or respiratory disease that, together with unclean fuel use, compound the risk of stillbirth (25). These environmental exposures—often invisible in clinical assessments—could convey a broader physiological burden that increases the risk of poor pregnancy outcomes.

Any alcohol use in the past month was associated with a higher odds of ever having experienced a stillbirth (AOR = 1.43; 95% CI: 1.06–1.93; *p* = 0.0195). This finding is consistent with prior research demonstrating that maternal alcohol use elevates stillbirth risk via multiple physiological pathways (62, 63). Alcohol can directly injure the fetus and placenta, and heavy prenatal alcohol use has been linked to fetal growth restriction, placental abruption, and fetal death (63). Combined prenatal drinking and smoking has also been shown to increase the odds of stillbirth nearly threefold, underscoring how alcohol may amplify the harm of smoke-borne toxins common to both tobacco and biomass combustion (64, 65). Beyond its physiological impacts, alcohol use may serve as a behavioral marker of reduced engagement in health-promoting practices, such as transitions to cleaner cooking methods (66). That is, alcohol use could compete with cleaner fuel adoption, either directly through financial diversion or indirectly through reduced maternal prioritization of environmental health (66, 67).

### 4.3 Integrating environmental risk screening into maternal health services

Our results highlight that stillbirth in Ghana is a multifactorial public health issue, arising from an interplay between environmental exposures and maternal characteristics. Traditionally, antenatal screening and prenatal care protocols in Ghana focus solely on medical and obstetric risk factors like diabetes, hypertension, or prior obstetric history (68). Our findings suggest that questions about a woman's household environment —specifically, her cooking fuel type and kitchen ventilation practices— may help to integrate environmental exposures into a more holistic approach to prenatal care. For example, it may prove beneficial for maternal healthcare providers to routinely ask pregnant women about their primary cooking fuel type and where they cook as part of the intake history. This type of screening may help to flag women at higher risk of stillbirth due to household air pollution.

In resource-limited communities, counseling delivered by community health workers (CHW), especially women from the communities they serve, may be another culturally grounded way to promote healthier behaviors around both fuel use and pregnancy health. For instance, if a pregnant woman is found to be cooking indoors with solid fuels, health workers can counsel her on strategies to potentially mitigate smoke exposure, such as improving airflow, keeping children away from the cooking area, or switching to a cleaner fuel if possible. However, when cleaner fuels are unaffordable or unavailable, ANC and CHW programs may promote cooking outdoors, including in covered outdoor shelters or shared community cooking spaces. This low-cost approach is quick to implement; yet, feasibility may be limited by several barriers. For instance, prolonged seasonal rains could render open cooking areas unusable (69, 70), and safety and privacy concerns might deter women from cooking outside after dark (71–73). Ingrained cooking traditions that favor indoor solid fuel use may further limit willingness to shift cooking outdoors (72, 74). Programs should assess these barriers locally and co-design solutions to these barriers in partnership with women's groups and community leaders.

In areas where clean fuel programs or subsidies do exist, antenatal clinics might serve as a point of referral —connecting expectant mothers with community initiatives that provide improved cookstoves, LPG cylinder exchanges, or solar cookers. Integrating such environmental health checks into maternal services would require some training and awareness-raising among healthcare staff, but would align with a more holistic approach to female reproductive care. Ghanaian women, who overwhelmingly bear the burden of household cooking, are disproportionately exposed to the harms of indoor air pollution (29). Therefore, protecting pregnant women from harmful environmental exposures in the home should be seen as part of the continuum of prenatal care, just as important as providing iron supplements or blood pressure monitoring (75). Any intervention aiming to reduce stillbirth should therefore be attuned to the lived realities of women's household cooking roles.

### 4.4 Future research directions

While this analysis leveraged nationally representative survey data, it relied on proxy measures (e.g., cooking fuel type) to estimate exposure to household air pollution. To clarify temporality and evaluate potential causal pathways, future prospective longitudinal studies in Ghana should incorporate direct environmental measurements. One priority is the in-home monitoring of indoor air quality in Ghanaian households — for instance, using portable monitoring devices to measure PM₂.₅ and CO levels during cooking hours. Such exposure measurements would enable dose-response analyses to determine how much reduction in indoor air pollution is needed to lower stillbirth risk meaningfully. It might also confirm whether unclean fuel use is a valid proxy for indoor air pollution, especially considering factors like stove efficiency and household ventilation.

The role of ventilation and housing design in modifying exposure also warrants closer investigation. Even when cleaner fuels are not immediately available, interventions like cooking outdoors or installing chimneys can drastically reduce indoor pollutant concentrations (41). Studies that document kitchen characteristics (i.e., outdoor vs. indoor kitchen, presence of windows, use of ventilation hoods) could assess how these features interact with cooking fuel type to influence stillbirth outcomes. Such investigations may guide interim risk reduction strategies, especially given that policy implementation can take up to 20 years to enact (76). Simple architectural modifications in homes can substantially lower smoke accumulation and toxin exposure (41). Urban planners and architects could incorporate ventilation considerations into housing programs, ensuring that low-cost homes have provisions for safe cooking. Similarly, development projects might include community cooking centers that serve multiple households.

In parallel, implementation research is needed to address sociocultural and structural barriers to clean fuel adoption. Although clean cooking technologies exist, uptake remains low because those at highest risk of household air pollution exposure cannot access or afford these newer appliances (77, 78). Additionally, cultural preferences, perceived safety concerns, and cost-related obstacles all contribute to continued reliance on solid fuels (72–74, 77, 79). For example, some households report that traditional foods taste better when cooked over open flames or that LPG refills are expensive and difficult to access (32).

Future research should delve into these barriers by engaging with the community through interviews, focus groups, and behavioral trials to design interventions that are culturally acceptable and address practical constraints. At the forefront of these efforts, local cooking habits should be readily accommodated, and cooking devices must be adapted in a user-friendly, culturally sensitive way. Involving women in Ghana in this process is crucial to ensure that the technology is adopted and yields its intended benefits. This could include exploring micro-financing schemes for LPG purchases or community education to dispel myths about new stove technologies. Multidisciplinary research —combining environmental science, public health, and social science— is needed to translate these epidemiological findings into effective stillbirth prevention strategies on the ground.

### 4.5 Strengths and limitations

By drawing on the 2022 Ghana Demographic and Health Survey, we analyzed a large, nationally representative sample of reproductive-aged women, yielding stillbirth estimates that carry direct relevance for current policy discussions. Our survey-weighted analytic framework accounted for clustering and stratification. Further, multiple imputation for missing BMI values mitigated potential selection bias. All covariates were selected *a priori* based on biological and epidemiological relevance, which reinforces the internal validity of our adjusted estimates.

However, several limitations must be acknowledged. First, the cross-sectional design precludes establishing temporality; findings indicate associations rather than causal effects, and reverse causation cannot be excluded.

With regard to our primary outcome of ever-stillbirth, it is important to note that stillbirth classification in the DHS relies on maternal recall of gestational age (in months) and birth outcome (34). This introduces the risk of (a) misclassification around the ≥7-month threshold (e.g., late miscarriages recorded as stillbirths or vice versa) and (b) confusion between stillbirths and early neonatal deaths. These issues characteristic of population survey data are well documented (1, 7). In addition, under-reporting of stillbirths —potentially due to stigma or grief— may bias actual ever-stillbirth prevalence downward; however, the direction of bias is uncertain until further studies are conducted.

Another limitation is with respect to exposure measurement. We used the household's primary type of cooking fuel as a proxy for household air pollution. The DHS does not record in-home PM₂.₅ and CO levels, stove efficiency, indoor cooking layout, or kitchen ventilation practices. These unmeasured covariates can critically moderate a woman's actual exposure to indoor air pollutants. For example, it is possible that some women who use LPG may also burn charcoal at times, or some women who use solid fuels may cook outdoors or have well-ventilated kitchens. This introduces the potential for exposure misclassification, potentially diluting the association and pushing estimates toward the null. In addition, the primary fuel type was missing for <2% of respondents, with slightly higher missingness among younger women and among those reporting a prior stillbirth. Although the proportion is small, our complete-case approach may introduce minor selection bias.

This study design also makes our findings susceptible to residual and unmeasured confounding. That is, the DHS does not capture several clinically important covariates, such as maternal anemia, hypertensive disorders in pregnancy, and gestational diabetes. Omission of these factors could lead to residual confounding and may bias estimates toward or away from the null. Future studies should measure these variables directly.

Other key covariates included in our final multivariable model, like alcohol intake and self-rated health, were self-reported and may be subject to social-desirability bias.

The exclusion of several important maternal healthcare variables is another limitation. The number of antenatal care visits, place of delivery, and caesarean section were missing for >50% of our analytic sample because the DHS collects them only for pregnancies within the five years preceding the survey. Because this missingness is not at random, incorporating these variables would have required restricting the analysis to pregnancies within the five years preceding the survey. That restriction would both reduce our sample size and alter our outcome from “ever-stillbirth” over the life course to stillbirths occurring within a five-year window prior to the survey. Pregnancy recency is also correlated with maternal age, parity, and health-seeking behavior; conditioning on it could introduce selection bias and yield estimates that are not comparable with those based on lifetime reproductive history. Accordingly, we did not perform a restricted-sample sensitivity analysis. Instead, we summarized these variables descriptively and excluded them from bivariate screening and multivariable models, acknowledging that their omission may introduce omitted-variable bias that could either over- or under-estimate the association between fuel type and stillbirth.

Finally, while our findings are broadly consistent with work from other LMICs, cultural and infrastructural differences may limit generalizability beyond Ghana. Due to these limitations, we recommend that policy makers interpret these estimates with caution.

## 5 Conclusion

In conclusion, this study provides evidence that unclean cooking fuel use may be associated with stillbirth in Ghana, even after adjusting for key covariates. While Ghana has made significant headway in reducing stillbirth, further reductions may be supported by strategies that reduce household air pollution —alongside improvements in clinical care. Integrating environmental risk checks within antenatal services may be a pragmatic step while stronger evidence accumulates. Because these data cannot establish temporality or causation, prospective longitudinal studies are needed. Those studies should include direct household air pollution measurements (e.g., PM₂.₅, CO) and richer clinical and environmental covariates to clarify pathways and to estimate the potential impact of clean-cooking interventions on stillbirths in Ghana.

## Data Availability

The Demographic and Health Survey datasets analyzed in this study are publicly available from The DHS Program (https://www.dhsprogram.com) upon registration and approval. The 2022 Ghana IR (women's) file (filename: GHIR8BFL) can be requested from the DHS Program microdata repository. Additionally, the analysis code used in this study is available from the corresponding author upon reasonable request. No DHS data can be shared without prior approval and access is via The DHS Program as described above.
